# A Fetal Electrocardiogram Signal Extraction Algorithm Based on the Temporal Structure and the Non-Gaussianity

**DOI:** 10.1155/2016/9658410

**Published:** 2016-01-26

**Authors:** Yibing Li, Wei Nie, Fang Ye, Ao Li

**Affiliations:** ^1^College of Information and Communication Engineering, Harbin Engineering University, Harbin 150001, China; ^2^School of Computer Science and Technology, Harbin University of Science and Technology, Harbin 150080, China

## Abstract

Fetal electrocardiogram (FECG) extraction is an important issue in biomedical signal processing. In this paper, we develop an objective function for extraction of FECG. The objective function is based on the non-Gaussianity and the temporal structure of source signals. Maximizing the objective function, we can extract the desired FECG. Combining with the solution vector obtained by maximizing the objective function, we further improve the accuracy of the extracted FECG. In addition, the feasibility of the innovative methods is analyzed by mathematical derivation theoretically and the efficiency of the proposed approaches is illustrated with the computer simulations experimentally.

## 1. Introduction

In biomedical signal processing, fetal electrocardiogram (FECG) extraction is full of challenges. FECG provides important information about the health of the fetus. However, FECG is always buried in various interferences and noises. Among these interferences and noises, maternal electrocardiogram (MECG) is the strongest one with high amplitude. Besides, breathing artifact, electrode artifact, and other noises also affect the desired FECG. Therefore, it is a difficult task to extract accurate FECG.

Traditional methods cannot get satisfactory results, such as multireference adaptive noise cancellation [1] and singular value decomposition [2]. Recently, the blind source separation (BSS) model [3–6] is introduced to solve the extraction problem of the desired FECG and shows satisfactory results. BSS algorithms can separate all sources from mixtures without any priori knowledge. In BSS algorithms, the ICA model [7–9] utilizes the non-Gaussianity [10] of signals to separate all source signals. This model is suitable for biomedical signal processing [11] and non-Gaussianity becomes an important tool to process these kinds of signals. In [12], traditional BSS algorithms and joint BSS algorithms were used to separate the maternal signal and the fetal signal. Authors [13] proposed two methods based on hybrid BSS to get FECG signals. These methods get better results than traditional BSS algorithms. However, the extraction of the desired FECG with the BSS algorithms is not the best choice. On one hand, separating all sources is a waste of time and not necessary if only the desired FECG is needed. On the other hand, the prior knowledge about the desired FECG can be utilized by us. According to this situation, blind source extraction (BSE) rises in response to the proper time and conditions and becomes a better choice than BSS. BSE only separates one source signal every time, so it has higher efficiency when it extracts the desired FECG. FECG is a special signal with the temporal structure. As prior knowledge, this structure can help us recover source signals. Barros and Cichocki [14] proposed a BSE algorithm called BCBSE. This algorithm can extract the desired FECG by utilizing the temporal structure. However, the algorithm is very sensitive to the estimation error of the optimal time delay. To overcome the drawback of BCBSE, Shi and Zhang [15] developed another BSE algorithm called SemiBSE based on the non-Gaussianity and the mean squared error function that is described in [14]. This method improves the performance of BCBSE. It is more robust for the estimation error of the optimal time delay. However, the performance of this algorithm is up to the choice of the parameters. If we initialize the parameters randomly, the performance of the algorithm becomes weaker. A BSE method called MACBSE [16] was proposed based on several time-delay autocorrelations of primary sources. MACBSE adopts a fixed-point learning algorithm for extraction of the desired source signal without choosing the learning step size. Authors [17] proposed a BSE algorithm called GABSE based on the generalized linear or nonlinear autocorrelations of the sources. This method has fast convergence speed and good stability if any two sources are uncorrelated with each other and have different temporal structures. A fast and robust fixed-point algorithm based on the nonlinear autocorrelation was proposed in [18]. Its convergence speed is shown to be at least quadratic. Li and Liao [19] proposed an algorithm based on the eigenvalue decomposition of the cross-correlation of whitened source signals at a given time tag. This algorithm functions without iterations. Wang et al. [20] proposed a robust separation algorithm to recover original sources through a joint diagonalizer of several average delayed covariance matrices at positions of the optimal time delay and its integers.

In order to settle the above problem better, we put forward the method based on the non-Gaussianity and the temporal structure of source signals. This paper consists of two main parts. First, we design an objective function to get the weight vector for extraction of the desired FECG. Then, the weight vector obtained by maximizing the objective function is combined with the FastICA algorithm to further improve the performance of the algorithm.

This paper is organized as follows.  Section 2 introduces the basic theory of our algorithms. As the principle part, we highlight the algorithms and the analysis about the algorithms in Section 3. Simulation results are presented in Section 4, and conclusions are made in Section 5.

## 2. The Basic Model

The linear instantaneous mixed model of BSS problems can be denoted as

(1) $$\begin{array}{c} x\left(\;t\;\right)=As\left(\;t\;\right)+n\left(\;t\;\right), \end{array}$$

where **x**(*t*) = [*x*_1_(*t*), *x*_2_(*t*),…, *x*_*n*_(*t*)]^*T*^ is the *n*-dimension mixture vector, **A** = [**a**_1_,…, **a**_*m*_] is an unknown mixing matrix, **s**(*t*) = [*s*_1_(*t*), *s*_2_(*t*),…, *s*_*m*_(*t*)]^*T*^ is the *m*-dimension source vector, and **n**(*t*) is the noise vector. If the noise is ignored, the noiseless model of BSS problems can be expressed as

(2) $$\begin{array}{c} x\left(\;t\;\right)=As\left(\;t\;\right). \end{array}$$

Preprocessing for the mixtures is usually necessary before the BSS algorithms. First, the mixtures are made to own zero mean through the following formula:

(3) $$\begin{array}{c} \hat{x}\left(\;t\;\right)=x\left(\;t\;\right)-E\left[\;x\left(\;t\;\right)\;\right], \end{array}$$

where $\hat{x}(t)$ is the new mixture vector. Then, the prewhitening process makes any two variables of the new mixtures orthogonal and gets the whitened mixtures

(4) $$\begin{array}{c} \tilde{x}\left(\;t\;\right)=V\hat{x}\left(\;t\;\right), \end{array}$$

where **V** is the prewhitening matrix. For a BSE algorithm, only a source signal can be separated at a time. If the unmixing matrix is **w** = (*w*_1_, *w*_2_,…, *w*_*n*_)^*T*^, the estimated source signal can be denoted as

(5) $$\begin{array}{c} y\left(\;t\;\right)=w^{T}\tilde{x}\left(\;t\;\right). \end{array}$$

Meanwhile, the delayed estimated source signal can be expressed as

(6) $$\begin{array}{c} y\left(\;t-\tau \;\right)=w^{T}\tilde{x}\left(\;t-\tau \;\right), \end{array}$$

where *τ* is the time delay that is some lag constant. The estimation error of the optimal time delay may have bad effect on the performance of the algorithms, so the reasonable estimation is important. The specific estimation method of the optimal time delay refers to [14].

## 3. The Proposed Algorithms

### 3.1. The Proposed Algorithm 1

#### 3.1.1. Objective Function

In order to avoid the blindness of choosing parameters, we combine the non-Gaussianity and the temporal structure to design the following constrained maximization problem:

(7) maxw=1 ψw=EGytGytyt−τ=EGwTx~tGwTx~tx~t−τTw,

where *G* is a differentiable nonlinear function. This function utilizes the non-Gaussianity, the temporal structure, and the nonlinear correlation.

#### 3.1.2. Learning Algorithm

With respect to **w**, the gradient of *E*{*G*[*y*(*t*)]*G*[*y*(*t*)*y*(*t* − *τ*)]} can be obtained as

(8) ∂EGytGytyt−τ∂w=Ex~tgytGytyt−τ+Ex~tyt−τgytyt−τGyt+Ex~t−τytgytyt−τGyt,

where *g* is the derivative of *G*. According to the gradient ascent learning rule, a gradient method can be derived as follows:

(9) w⟵w+μ∂EGytyt−τ∂w=w+μEx~tgytGytyt−τ+Ex~tyt−τgytyt−τGyt+Ex~t−τytgytyt−τGyt,w=ww,

where *μ* is a learning rate. The desired vector can be obtained through this method, but the corresponding cost is that the convergence speed of the algorithm is slow and the performance of the algorithm is dependent on the proper choice of the learning rate. If the learning rate is chosen improperly, the convergence property will be destroyed. Therefore, how to find a new approach to radically improve the convergence speed and the reliability is an important issue that needs to be solved. The fixed-point algorithm is a choice to solve this issue. For getting a more efficient fixed-point algorithm, we note that the gradient must point in the direction of **w** at a stable point of the gradient algorithm. It means the gradient must be equal to **w** multiplied by some scalar constant. In such a case, adding the gradient to **w** does not change its direction and the convergence can be obtained. Through normalization to the unit norm, the value of **w** is not changed except by changing its sign. Therefore, the following formula can be obtained:

(10) w∝Ex~tgytGytyt−τ+Ex~tyt−τgytyt−τGyt+Ex~t−τytgytyt−τGyt.

Through the above formula, the fixed-point algorithm can be updated as

(11) w⟵Ex~tgytGytyt−τ+Ex~tyt−τgytyt−τGyt+Ex~t−τytgytyt−τGyt,w=ww.

Utilizing the above fixed-point algorithm, we can extract the desired signal.

#### 3.1.3. Stability Analysis

In this part, we analyze the stability of the proposed algorithm.

**Theorem 1 .**

Assume that the input data meet the model as (2). The data are prewhitened through equation $\tilde{x}=VAs$ and *G* is a quite smooth even function. Furthermore, we assume that *δs*_*i*_ = *s*_*i*_(*t*)*s*_*i*_(*t* − *τ*)  (*i* = 1,2,…, *n*) are mutually independent. To simplify it, *s*_*i*_(*t*)*s*_*i*_(*t* − *τ*) is replaced by *s*_*i*_ *s*_*iτ*_. Under the constraint ‖**w**‖ = 1, the local maxima of *E*{*G*[*y*(*t*)]*G*[*y*(*t* − *τ*)]} include one row of the inverse of the matrix **V** **A** if the corresponding desired signal *s*_*i*_ satisfies

(12) Eg′siGsisiτ+siτgsigsisiτ+2si2g′sisiτGsi+2sisiτsjsjτg′sisiτGsi+3sisjsjτgsigsisiτ−sigsiGsisiτ−2sisiτgsisiτGsi<0∀i≠j,

where *g*′ is the derivative of *g*.

**Proof**

According to the above conditions, we make the orthogonal transform of coordinates **p** = **A** ^*T*^ **V** ^*T*^ **w** and obtain

(13) $$\begin{array}{c} H\left(\;p\;\right)=E\left\{\;G\left(\;p^{T}s{s_{\tau }}^{T}p\;\right)\;\right\}. \end{array}$$

We analyze the stability at the point **p** = **e**_1_ = (1,0, 0,0,…)^*T*^. The independency assumptions are utilized to evaluate the gradient and the Hessian matrix of *H*(**p**) at the point **p** = e_1_. Then, through making a small perturbation *ɛ* = (*ɛ*_1_, *ɛ*_2_,…), where *ɛ*_1_ and *ɛ*_2_ are the elements of *ɛ*, we get

(14) He1+ɛ=He1+ɛT∂He1∂p+12ɛT∂He1∂pɛ+oɛ2,

where ∂*H*(**e**_1_)/∂**p** and ∂^2^ *H*(**e**_1_)/∂**p** ^2^ are expressed as

(15) ∂He1∂p=e1Es1gs1Gs1s1τ+2s1s1τgs1s1τ·Gs1,∂H2e1∂p2=diag⁡Es12g′s1Gs1s1τ+4s12s1τgs1gs1s1τ+4s12s1τ2g′s1s1τGs1,Eg′s1Gs1s1τ+s1τgs1gs1s1τ+2s12g′s1s1τGs1+2s1s1τs2s2τg′s1s1τGs1+3s1s2s2τgs1gs1s1τ,…,Eg′s1Gs1s1τ+s1τgs1gs1s1τ+2s12g′s1s1τGs1+2s1s1τsjsjτg′s1s1τGs1+3s1sjsjτgs1gs1s1τ.

Because of the constraint ‖**w**‖ = 1, we can know ${ɛ}_{1}=\sqrt{1-{ɛ}_{2}^{2}-\cdots }-1$. Meanwhile, $\sqrt{1-\gamma }=1-\gamma /2+o(\gamma )$ is known. The order of *ɛ*_1_^2^ is *o*(‖*ɛ*‖^2^), so the higher order terms can be neglected. Through the first-order approximation of *ɛ*_1_ that is described above, we know *ɛ*_1_ = −∑_*i*>1_ *ɛ*_*i*_^2^/2 + *o*(‖*ɛ*‖^2^) and obtain

(16) He1+ɛ=He1+12Eg′s1Gs1s1τ+s1τgs1gs1s1τ+2s12g′s1s1τGs1+2s1s1τsjsjτg′s1s1τGs1+3s1sjsjτgs1gs1s1τ−s1gs1Gs1s1τ−2s1s1τgs1s1τGs1∑j>1ɛj2+oɛ2.

**p** = **e**_1_ is an extremum that is implied in the condition of Theorem 1 if the following condition is satisfied:

(17) Eg′s1Gs1s1τ+s1τgs1gs1s1τ+2s12g′s1s1τGs1+2s1s1τsjsjτg′s1s1τGs1+3s1sjsjτgs1gs1s1τ−s1gs1Gs1s1τ−2s1s1τgs1s1τGs1<0.

Based on the above descriptions, this condition can be expanded as

(18) Eg′siGsisiτ+siτgsigsisiτ+2si2g′sisiτGsi+2sisiτsjsjτg′sisiτGsi+3sisjsjτgsigsisiτ−sigsiGsisiτ−2sisiτgsisiτGsi<0∀i≠j.

#### 3.1.4. Convergence Analysis

Convergence is also important like stability. In this part, we analyze the convergence of the algorithm.

**Theorem 2 .**

If the following two conditions are satisfied, the algorithm described in formula (11) can reach convergence:

(1) {*s*_*i*_, *s*_*iτ*_} and {*s*_*j*_, *s*_*jτ*_} are mutually independent.
(2) *E*{*s*_*i*_ *g*(*s*_*i*_)*G*(*s*_*i*_ *s*_*iτ*_) + 2*s*_*i*_ *s*_*iτ*_ *g*(*s*_*i*_ *s*_*iτ*_)*G*(*s*_*i*_)} ≠ 0.

**Proof**

Based on the orthogonal coordinate transformation **p** = **A** ^*T*^ **V** ^*T*^ **w**, formula (11) is changed to

(19) p^k=EsgpTksGpTkssτTpk+EspTksτgpTkssτTpkGpTks+EsτpTksgpTkssτTpkGpTks,pk+1=p^kp^k,

where *k* is the number of iterations. Using a Taylor approximation for *G* and *g*, we have

(20) GpTks=Gpisi+gpisip−iTs−i+op−i2,gpTks=gpisi+g′pisip−iTs−i+op−i2,GpTkssτTpk=Gpisipisiτ+gpisipisiτp−iTs−is−iτTp−i+op−i2,gpTkssτTpk=gpisipisiτ+g′pisipisiτp−iTs−is−iτTp−i+op−i2,

where **p**_−*i*_ is the vector **p** without its *i*th component, **s**_−*i*_ is the vector **s** without its *i*th component, and **s**_−*iτ*_ is the vector **s**_*τ*_ without its *i*th component.

Similarly, we analyze the convergence at the point **p** = **e**, where *e*_*i*_ is 1 and *e*_*j*_ is 0 (∀*j* ≠ *i*). Based on the above conditions, we get

(21) p^i=EsigsiGsisiτ+2sisiτgsisiτGsi+op−i2,p^j=Eg′siGsisiτ+siτgsigsisiτ+sisjsjτgsigsisiτpj+op−i2.

The above equations clearly show that the algorithm converges to such a vector **e** in which *e*_*i*_ is 1 and *e*_*j*_ is 0 if condition (2) is satisfied. It means that *w* = ((**V** **A**)^*T*^)^−1^ **q** can converge without computing the sign. This shows that **w** converges as Theorem 2.

### 3.2. The Proposed Algorithm 2

On the basis of the above algorithm, we propose another improved algorithm by utilizing the FastICA algorithm. The FastICA algorithm is a typical ICA algorithm. The source signal can be estimated through maximizing the non-Gaussianity function of signals. One of the representative non-Gaussianity functions is negentropy. However, this algorithm is based on the theory of projection pursuit, so any signal may be first extracted. According to this problem, we propose an improved algorithm by presetting the weight vector. The method of projection pursuit searches for a projection output to maximize the objective function. Based on the theory of ICA, a local extremum in the solution space of the projection direction is obtained if the negentropy is the objective function. The output of the corresponding data for this local extremum is an independent component. If the mixtures consist of *m* source signals, the parameter space formed by ICA will have 2*m* local extrema that correspond to signed solutions of all source signals. Meanwhile, a local optimal solution owns its attraction region for a greedy optimization algorithm that is based on projection pursuit. In other words, the initial point in this region converges to this local optimal solution with this algorithm. The FastICA algorithm may not extract the desired signal if the initial weight matrix is arbitrary. However, if the initial weight matrix is near the local optimal solution that corresponds to the desired signal, the desired source signal can be extracted. The solution vector of the proposed algorithm 1 can be the initial weight matrix of the FastICA algorithm and help the algorithm extract the desired source signal. The solution vector of the proposed algorithm 1 is denoted as

(22) $$\begin{array}{c} w_{0}={\left[\;w_{1},\ldots ,w_{i},\ldots ,w_{j},\ldots ,w_{n}\;\right]}^{T}. \end{array}$$

It is the initial weight matrix of the proposed algorithm 2. Then, we can extract the desired signal with the FastICA algorithm based on the negentropy. The approximate negentropy in literatures can be denoted as

(23) $$\begin{array}{c} J\left(\;y\;\right)\propto {\left\{\;E\left[\;G\left(\;y\;\right)\;\right]-E\left[\;G\left(\;v\;\right)\;\right]\;\right\}}^{2}. \end{array}$$

The gradient of *J*(*y*) is written as

(24) $$\begin{array}{c} \nabla J\left(\;y\;\right)\propto \gamma E\left\{\;\tilde{x}\left(\;t\;\right)g\left[\;y\left(\;t\;\right)\;\right]\;\right\}, \end{array}$$

where *γ* = *E*[*G*(*y*)] − *E*[*G*(*v*)]; the learning algorithm is obtained as follows:

(25) Δw=EGy−EGvEx~tgyt,w⟵ww.

The corresponding fixed-point algorithm is denoted as

(26) w⟵Ex~tgyt,w⟵ww.

However, the convergence property of the FastICA algorithm based on the negentropy is unsatisfactory because the nonpolynomial moment does not own good algebra property. Therefore, the Newton iteration algorithm is usually utilized to improve the process. If the maximization of *E*{*G*[**w** ^*T*^ **x**(*t*)]} under the constraint ‖**w**‖ = 1 is considered, the optimization problem with the Lagrangian multiplier method can be changed to

(27) $$\begin{array}{c} F\left(\;w\;\right)=E\left\{\;G\left[\;w^{T}\tilde{x}\left(\;t\;\right)\;\right]\;\right\}+\beta w^{T}w. \end{array}$$

The first-order derivative and the second-order derivative can be denoted as

(28) ∂Fw∂wEx~tgwTx~t+2βw,∂2Fw∂w2Ex~tx~tTg′wTx~t+2βI≈Ex~tx~tTEg′wTx~t+2βI=Eg′wTx~tI+2βI.

In order to avoid calculating the inverse of the matrix, the simple approximation is made to get the following Newton iteration algorithm:

(29) ww−∂Fw/∂w∂2Fw/∂w2=w−Ex~tgwTx~t+2βwEg′wTx~t+2β.

Both sides of the above equation are multiplied by $E\{g^{\prime }[w^{T}\tilde{x}(t)]\}+2\beta$ and then the learning algorithm after the algebraic simplification is denoted as

(30) w⟵Ex~tgwTx~t−Eg′wTx~tw,w⟵ww.

Formula (30) is the basic formula of the fixed-point FastICA algorithm.

Here, we analyze the condition that the initial weight vector must meet in order to get the desired signal. According to formula (30), we get

(31) w^k⟵Ex~tgwkTx~t−Eg′wkTx~twk,wk+1=w^kw^k.

Based on formula (31) and **q** = **A** ^*T*^ **V** ^*T*^ **w**, the following formula is obtained:

(32) q^k⟵EstgqkTst−Eg′qkTstqk,qk+1=q^kq^k.

Assuming $a_{k}=1/\left\|\hat{q}(k)\right\|$, we obtain

(33) qk+1=akEstgqkTst−akEg′qkTstqk.

To analyze conveniently, we choose *G* = *y* ^4^/4 to illustrate. Therefore, *g* = *y* ^3^ and *g*′ = 3*y* ^2^ can be obtained. Formula (33) can be simplified as

(34) qk+1=akEs1∑m=1Mqmksm3−3q1k·E∑m=1Mqmksm2,…,Esi∑m=1Mqmksm3−3qik·E∑m=1Mqmksm2,…,EsM∑m=1Mqmksm3−3qMk·E∑m=1Mqmksm2T.

Next, the following formula can be obtained:

(35) qik+1=akEsi∑m=1Mqmksm3−3akqikE∑m=1Mqmksm2=akEsiqiksi+∑m=1,m≠iMqmksm3−3akqikEqiksi+∑m=1,m≠iMqmksm2=akEqi3ksi4+3qiksi2∑m=1,m≠iMqmksm2−3akqikEqi2ksi2+∑m=1,m≠iMqmksm2=akqi3kEsi4−3akqi3kEsi2=akqi3kEsi4−3Esi2=akqi3kk4si,

where *k*_4_(·) denotes the kurtosis of the signal. Similarly, we obtain

(36) $$\begin{array}{c} q_{j}\left(\;k+1\;\right)=a_{k}{q_{j}}^{3}\left(\;k\;\right)k_{4}\left(\;s_{j}\;\right). \end{array}$$

The following formula is assumed:

(37) bk+1qjk+1qik+1=qj3kk4sjqi3kk4si=b3kk4sjk4si.

Then, *b*(*k*) can be denoted as

(38) bkqjkqik=k4sjk4si−1qj1qi1k4sjk4si3k−1.

If we want to get the desired signal, *q*_*j*_(*k*) must be close to 0 and *q*_*i*_(*k*) must be close to 1. Therefore, the following formula must be met:

(39) $$\begin{array}{c} \left|\;\frac{q_{j}\left(1\right)}{q_{i}\left(1\right)}\sqrt{\left|\frac{k_{4}\left(s_{j}\right)}{k_{4}\left(s_{i}\right)}\right|}\;\right|<1. \end{array}$$

The above formula (39) can be simplified as

(40) $$\begin{array}{c} {q_{i}}^{2}\left(\;1\;\right)>{q_{j}}^{2}\left(\;1\;\right)\left|\;\frac{k_{4}\left(s_{j}\right)}{k_{4}\left(s_{i}\right)}\;\right|. \end{array}$$

Combining the solution vector **w**_0_ described in formula (22), we get

(41) $$\begin{array}{c} q_{0}=A^{T}V^{T}w_{0}={\left[\;q_{01},\ldots ,q_{0i},\ldots ,q_{0j},\ldots ,q_{0n}\;\right]}^{T}. \end{array}$$

In conclusion, the desired signal can be obtained if the following condition is satisfied:

(42) $$\begin{array}{c} {q_{0i}}^{2}>{q_{0j}}^{2}\left|\;\frac{k_{4}\left(s_{j}\right)}{k_{4}\left(s_{i}\right)}\;\right|. \end{array}$$

Similarly, if another function *G* is chosen, there will be another condition that is similar to formula (42) helping us get the desired source signal.

## 4. Simulation Results and Analysis

### 4.1. Experiments on Artificial Data

**Figure 1:**
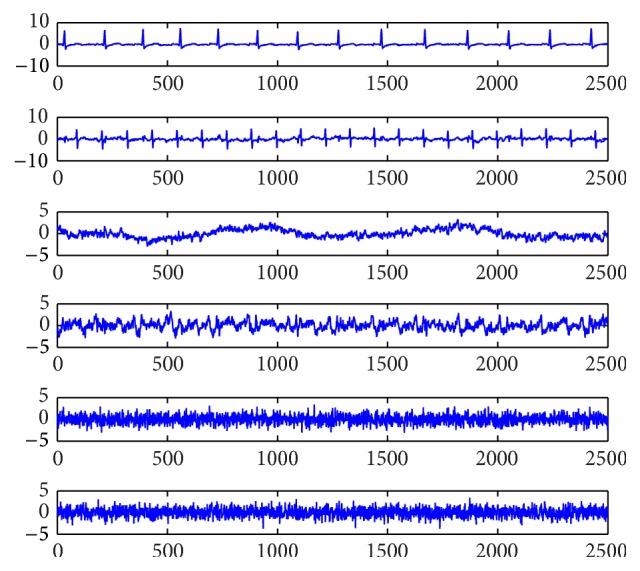
Source signals data.

**Figure 2:**
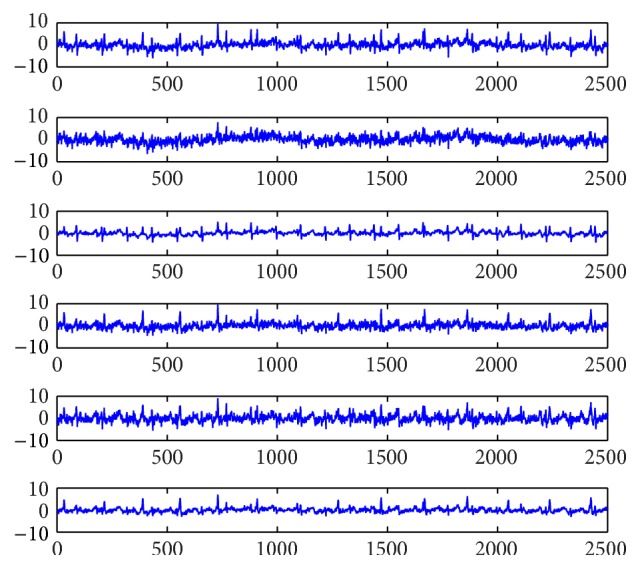
Mixed signals data.

In order to verify the performance of the above algorithms, simulation experiments are performed with the data that are shown in Figure 1. Source signals include maternal electrocardiogram (MECG), fetal electrocardiogram (FECG), breathing artifact, electrode artifact, and two kinds of noises. The above source signals are mixed with a matrix to get the mixtures that are shown in Figure 2. The mixing matrix in this paper is written as

(43) $$\begin{array}{c} \left[\begin{array}{cccccc} 0.8246 & 0.9469 & 0.7384 & 0.1109 & 0.5211 & 0.5746\\ 0.4530 & 0.5101 & 0.9764 & 0.3752 & 0.7743 & 0.8639\\ 0.3806 & 0.7919 & 0.5233 & 0.3299 & 0.1203 & 0.1986\\ 0.9259 & 0.4522 & 0.4299 & 0.3421 & 0.6255 & 0.6725\\ 0.7408 & 0.8492 & 0.2072 & 0.8171 & 0.3466 & 0.9018\\ 0.7376 & 0.3904 & 0.3234 & 0.5317 & 0.3346 & 0.1992 \end{array}\right]. \end{array}$$

Corresponding simulations are needed to compare the performance of the proposed algorithms and other algorithms. The performance of algorithms can be measured with the performance index (PI) that is denoted as

(44) $$\begin{array}{c} PI=\overset{n}{\underset{j=1}{\sum }}\frac{\left|p_{j}\right|}{\max_{k}\left|p_{k}\right|}-1,\;k=1,\ldots ,n, \end{array}$$

where *p*_*j*_ denotes the element of the global vector **p** = **w** ^*T*^ **V** **A**. If the desired signal is extracted perfectly, the value of PI is 0. In other words, the value of PI is lower if the performance is better. PI is always used to be the measurement index for the artificial data.

**Figure 3:**
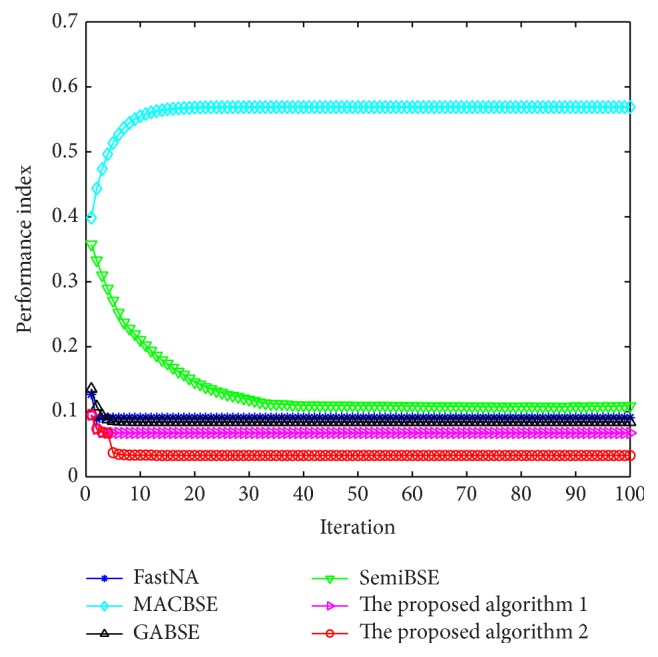
The performance indexes against iteration numbers by the six algorithms.

In the comparison algorithms, the MACBSE algorithm, the GABSE algorithm, the SemiBSE algorithm, and the FastNA algorithm are the algorithms that need iterations. Therefore, we compare these algorithms with the proposed algorithm through the relationship of the average performance index and the iterations. In this paper, the function *G*(*u*) = log⁡(cosh⁡(*u*)) is chosen. The parameters of other algorithms are selected based on the references. In the GABSE algorithm and the FastNA algorithm, *G*(*u*) = log⁡(cosh⁡(*u*)) is also chosen. In the SemiBSE algorithm, the nonlinearity is chosen as *g*(*u*) = sign⁡(*u*) and the constant coefficient is initialized to 0.3. The learning rates are set to 0.1 and 0.0001. All the algorithms described above adopt the same initial weight vector [−0.3331,0.3768,0.2715,0.6498,0.3576,0.3510]^*T*^ and the optimal time delay 112. The average performance indexes over 100 independent trials against iteration numbers by the six algorithms are shown in Figure 3.

It is obviously verified in Figure 3 that our algorithms have better performance than other algorithms when the algorithms achieve the convergence.

Meanwhile, we compare our algorithms with two noniterative algorithms which include Li's algorithm and the LAJD algorithm. In the LAJD algorithm, *L* and *K* are set to 3 and 2, respectively. The performance indexes of the algorithms after the convergence are shown in Table 1.

From Table 1, it is easy to know that our algorithms have lower performance indexes than Li's algorithm and the LAJD algorithm.

**Figure 4:**
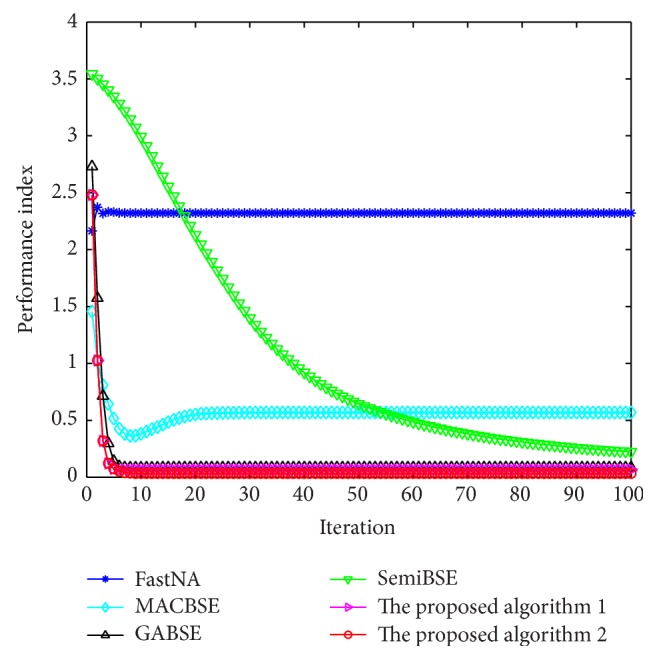
The average performance indexes in 100 independent trials against iteration numbers by the six algorithms when the mixing matrix is random.

The above comparison is done with the same mixing matrix. However, the mixing matrix is unknown and changing in practice. Therefore, Monte Carlo simulations need to be carried out with different random mixing matrices. We set the initial weight vector as [0,0, 0,0, 0,1]^*T*^ and compare different algorithms. The average performance indexes over 100 independent trials against iteration numbers by the six iterative algorithms are shown in Figure 4. In 100 independent trials, the mixing matrices are different and random.

When the mixing matrix is random, the average performance indexes of our algorithms and two noniterative algorithms in 100 independent trials are shown in Table 2.

As shown in Figure 4 and Table 2, our algorithms still own better performance when the mixing matrix is random.

Based on the comparisons of the proposed algorithms and all other algorithms, we can know that our algorithms own good performance for processing the artificial data.

### 4.2. Experiments on Real-World Data

**Figure 5:**
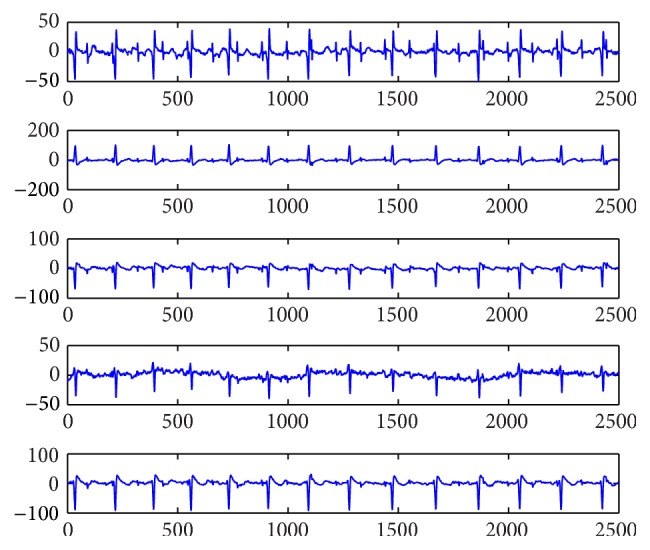
The real-world EEG data.

**Figure 6:**
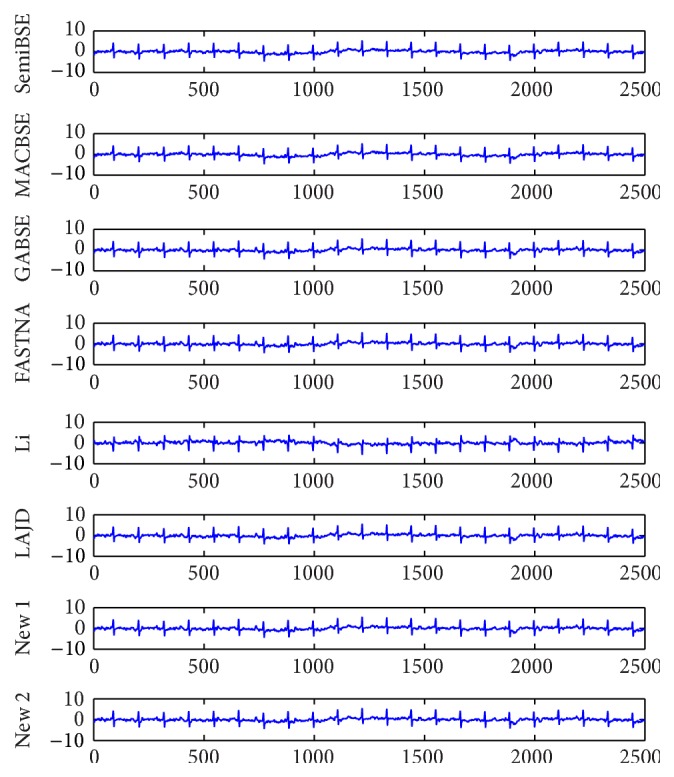
The FECG signals extracted by all algorithms at the optimal delay 112.

**Figure 7:**
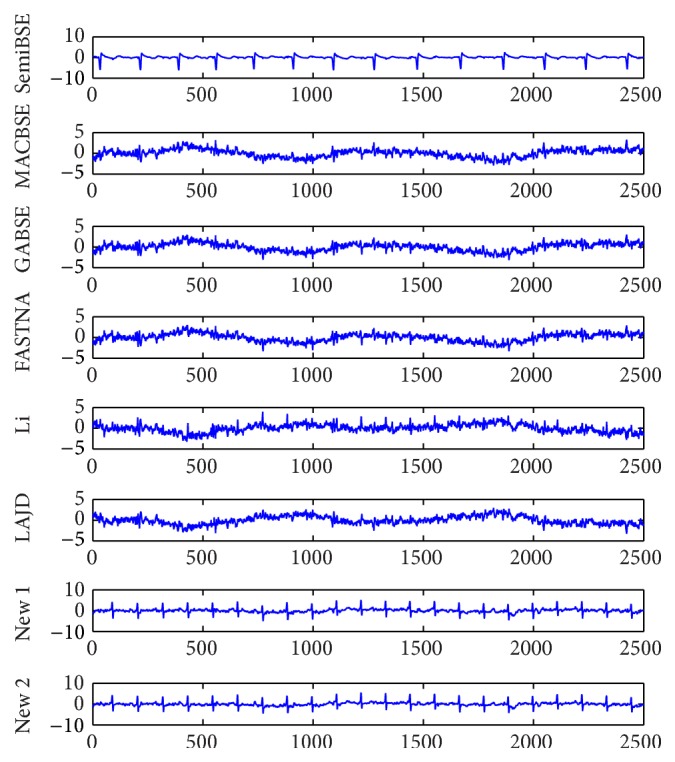
The FECG signals extracted by all algorithms at the optimal delay 106.

**Figure 8:**
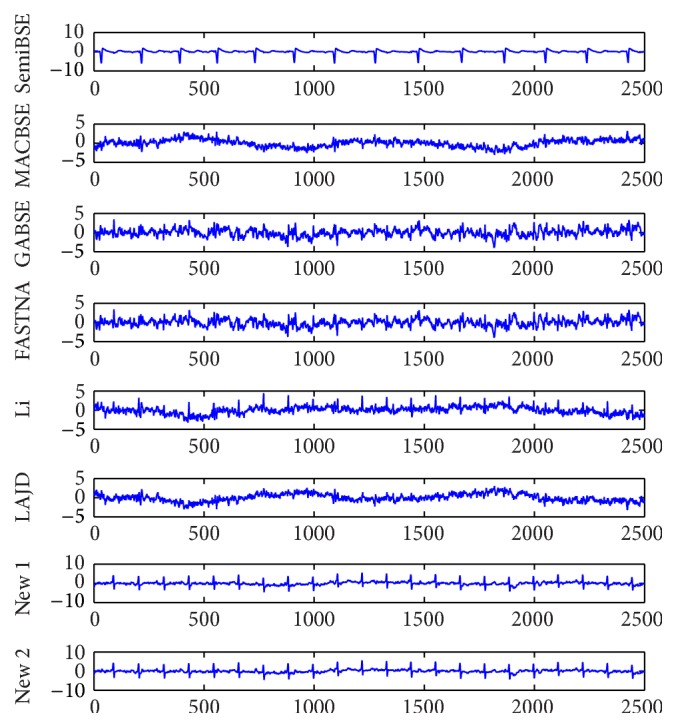
The FECG signals extracted by all algorithms at the optimal delay 120.

In order to further demonstrate the practicability of our algorithm, the simulations of real-world EEG data which are shown in Figure 5 need to be performed. The well-known ECG data are measured from a pregnant woman and shared by De Moor [21]. The ECG data are mixed with maternal electrocardiogram (MECG), fetal electrocardiogram (FECG), breathing artifact, electrode artifact, and some other noises. When extracting FECG from the real-world ECG signals with various kinds of algorithms, we cannot measure the performance of the algorithms with PI because the mixing matrix is unknown. However, the robustness of the algorithms is also an important index. It is always used to be the measurement index for the real-world data. Because there may be estimation error of the optimal time delay in practice, the robustness of the algorithms becomes more important. The FECG signals extracted by all algorithms at the optimal delay 112 are shown in Figure 6. The FECG signals extracted by all algorithms at delays 106 and 120 are shown in Figures 7 and 8. From Figure 6, we can find that all algorithms can extract the FECG signal at the optimal delay 112. However, if the estimation error of the delay is too large, the FECG may not be extracted by the algorithms. According to Figures 7 and 8, we can know that only the proposed algorithms can extract the FECG signal. Our algorithms function when the delay ranges from 106 to 120, which directly illustrates that our algorithms are more robust than other algorithms. In conclusion, our algorithm is applicable to real-world data and owns better robustness than other algorithms.

## 5. Conclusion

We have proposed novel BSE algorithms for extraction of FECG. First, we design an objective function based on the non-Gaussianity and the temporal structure of the desired FECG. Secondly, a clearer FECG can be obtained by the learning algorithm. Furthermore, the proposed algorithm is further improved through combining the vector obtained by the objective function with the FastICA algorithm. The algorithms of this paper are effective and robust. They can save more time and get more satisfactory results than traditional BSS methods through utilizing the prior information. Experimental results certify the effectiveness of the proposed algorithms. Due to the generality of the presented BSE algorithms, we believe that they can also be extended to other signal processing applications. With our algorithms, it is easy to design suitable functions to solve various kinds of problems.

## Figures and Tables

**Table 1:** The PI values of the algorithms.

Li's algorithm	The LAJD algorithm	Our algorithm 1	Our algorithm 2
0.2405	0.195	0.0673	0.033

**Table 2:** The average PI values of the algorithms when the mixing matrix is random.

Li's algorithm	The LAJD algorithm	Our algorithm 1	Our algorithm 2
0.2405	0.195	0.0672	0.033
